# *Davimacrocera*, a New Extant Genus of Keroplatidae, with Notes on the Fossil Genus *Burmacrocera* (Diptera)

**DOI:** 10.3390/insects15121018

**Published:** 2024-12-22

**Authors:** Jan Ševčík

**Affiliations:** 1 Department of Biology and Ecology, Faculty of Science, University of Ostrava, Chittussiho 10, CZ-710 00 Ostrava, Czech Republic; sevcikjan@hotmail.com; 2 Silesian Museum, Nádražní okruh 31, CZ-746 01 Opava, Czech Republic

**Keywords:** Bibionomorpha, Sciaroidea, fungus gnats, taxonomy, Oriental region, Burmese amber, Mesozoic

## Abstract

A new extant genus of Keroplatidae (Diptera: Bibionomorpha), *Davimacrocera* gen. nov., is described in this paper, which includes two new species, *Davimacrocera davidi* sp. nov. (Brunei, type species of the genus) and *D. taiwanensis* sp. nov. (Taiwan, China). Its relationships to other extant genera and possible phylogenetic position are briefly discussed, including a comparison with the fossil genus *Burmacrocera* Cockerell, 1917 from the mid-Cretaceous amber of Myanmar. The identity of *Burmacrocera* remains unclear, pending further studies of Keroplatidae in Burmese amber.

## Simple Summary

Nematocerous flies, or lower Diptera, are one of the most species-rich groups of insects. The family Keroplatidae belongs to the particularly speciose dipteran infraorder Bibionomorpha, of which larvae are mostly associated with fungi. The family is characterized by flattened antennae and bright coloration in some species, and in some genera, the larvae are known for their ability of bioluminescence. Although many new species and new genera have already been discovered and described in this family, many taxa still remain undescribed, especially outside Europe or North America, and one such genus is described as new to science in this paper. It is distributed in the Oriental region and shares interesting ancient features with some fossil representatives of the family.

## 1. Introduction

The family Keroplatidae represents one of the most diverse groups of fungus gnats (Diptera: Bibionomorpha: Sciaroidea), with about 1000 species described in almost 100 genera [1]. The phylogenetic relationships among the families and subfamilies of fungus gnats (Sciaroidea) have been broadly discussed in recent years, and several hypotheses have been proposed (e.g., [2,3,4]). Within the family Keroplatidae, six extant subfamilies are currently recognized [1], i.e., Arachnocampinae, Platyurinae, Macrocerinae, Sciarokeroplatinae, Lygistorrhininae, and Keroplatinae, plus an exclusively fossil one, Adamacrocerinae [5]. The former family Lygistorrhinidae was transferred to the family Keroplatidae as a result of a molecular study based on representative gene and taxon sampling [1], subsequently confirmed by a recent study by Lim et al. [6].

In the paper by Mantič et al. [1], an undescribed genus was included in the molecular phylogenetic tree, belonging to the well-supported clade “Macrocerinae sensu lato”, branching rather basally within the family and also comprising the subfamilies Macrocerinae and Sciarokeroplatinae. This undescribed extant genus was referred to as “genus near *Burmacrocera*”, indicating its morphological similarity with the fossil genus *Burmacrocera* Cockerell, 1917, known from the mid-Cretaceous amber of northern Myanmar (so-called Burmese amber). However, the identity and relationships of *Burmacrocera* have remained obscure, being placed either in Macrocerinae by the original author [7] or in the tribe Orfeliini of the subfamily Keroplatinae by some subsequent authors [8,9].

In this paper, the opportunity is taken to describe this new extant genus and discuss its relationships to other genera of fungus gnats, and to clarify the identity and phylogenetic position of *Burmacrocera*, based on the extensive collection of Burmese amber Sciaroidea, comprising more than 1200 specimens, accumulated during the last 6 years.

## 2. Materials and Methods

Specimens were examined using an SZX7 stereomicroscope (Olympus, Tokyo, Japan), equipped with an Olympus E-600 digital camera (Olympus, Tokyo, Japan), and an Olympus CX41 compound microscope (Olympus, Tokyo, Japan) equipped with an Olympus Infinity 1 digital camera (Olympus, Tokyo, Japan). Photographs were processed using Helicon Focus Pro 8 Software. The terminology principally follows that used in our recent papers on Keroplatidae [10,11], where the homology and wing vein nomenclature in Bibionomorpha were briefly explained.

The material was collected by the author with Malaise traps filled with 75% ethanol and is preserved in the following collections: Jan Ševčík Lab, University of Ostrava, Ostrava, Czech Republic (JSL-UOC); National Museum, Prague, Czech Republic (NMPC); National Museum of Natural Science, Taichung, Taiwan (NMNS); Silesian Museum, Opava, Czech Republic (SMOC); Universiti Brunei Darussalam, Brunei (UBDC). The holotypes are stored in pinned microvials filled with glycerol; the other specimens are in 75% ethanol.

This published work and the nomenclatural acts it contains have been registered in ZooBank, the online registration system for the ICZN. The LSID for this publication is urn:lsid:zoobank.org:pub:29BC2AFD-4C83-42CC-8238-FC1D7CF1AE33.

## 3. Results and Discussion

### 3.1. Description of a New Genus

#### 3.1.1. *Davimacrocera* gen. nov. (Figure 1 and Figure 2)

LSID: urn:lsid:zoobank.org:act:292A1A0D-5671-4346-83F8-66DAEC813CE2

**Type species.** *Davimacrocera davidi* sp. nov.

**Figure 1:**
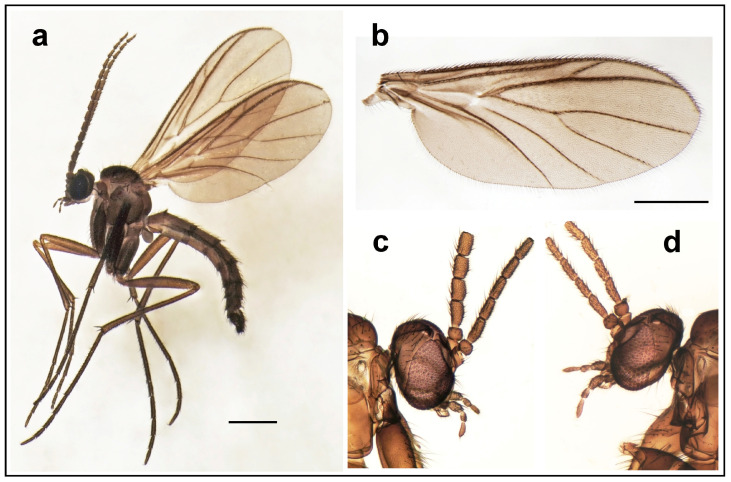
Habitus and wing venation of *Davimacrocera* gen. nov. (**a**) Habitus of *Davimacrocera taiwanensis* sp. nov. (holotype); (**b**) wing of *Davimacrocera davidi* sp. nov.; (**c**) head of *D. davidi* sp. nov. (male, holotype); (**d**) head of *D. davidi* sp. nov. (female, paratype). Scale bar = 0.5 mm.

**Diagnosis.** A relatively small (wing length 2.2–2.5 mm), delicate, brownish macrocerine fly (Figure 1a). Head with three ocelli, without distinct cerebral sclerite. Compound eye emarginated above the antennal base, forming an incomplete eye-bridge. Antennae with 14 cylindrical flagellomeres. Thoracic pleura and mediotergite bare. Wing (Figure 1b) relatively narrow, without markings and without macrotrichia on wing membrane. Sc short, ending in C, apically weak, not reaching to the base of Rs. Basal cell short, reaching to about one-fourth of the wing length. M-fork with a long stem, only slightly shorter than M_1_. Base of M_4_ weak and slightly approaching Cu. Anal vein short, distally weak. Legs and abdomen entirely dark brown. Tibial setulae arranged in longitudinal rows. Tibial spurs 1:2:2, about as long as tibial diameter. Terminalia with narrow gonostylus, slightly longer than gonocoxite.

**Figure 2:**
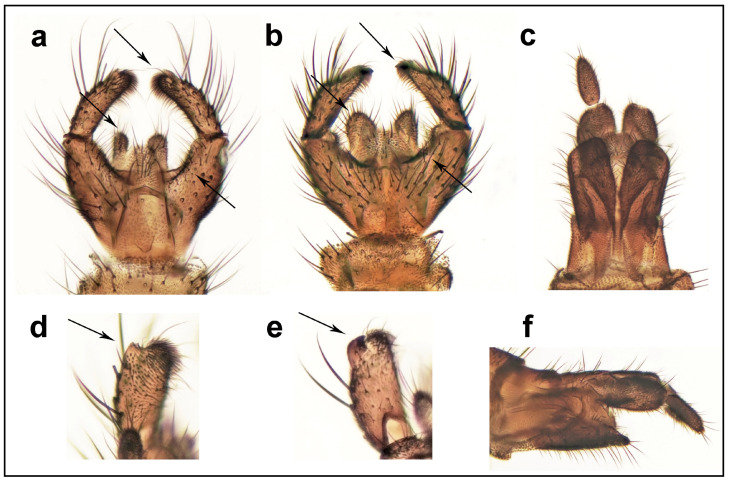
Male terminalia in ventral view, female terminalia in ventral view (right cercus missing), detail of gonostylus in mediodorsal view, and female terminalia in lateral view (in 100x magnification). (**a**,**c**,**d**,**f**) *Davimacrocera davidi* sp. nov. (holotype); (**b**,**e**) *D. taiwanensis* sp. nov. (holotype). The arrows indicate diagnostic characters for particular species.

**Description.** Male. Body all dark brown, including legs. Head with three ocelli closely set together, placed on anterior corner of subtriangular vertex (Figure 1c,d), but without distinct cerebral sclerite. Compound eye oval, distinctly emarginated above antennal base, forming an incomplete eye-bridge. Palpi with four palpomeres, plus palpiger basally. Antennae about 1.5 times as long as head and thorax put together, tapering, with 14 cylindrical flagellomeres, basal ones about as long as wide, apical ones about 1.5 times as long as wide. Each flagellomere has several dark erect setae, about as long as width of flagellomere. Scutum and scutellum with dark erect setae, about as long as width of scutellum. Thoracic pleura and mediotergite bare. Wing relatively narrow, with ratio of length to width about 3.1, its posterior margin proximally rounded, not angled. Wing veins covered with setae (macrotrichia) but wing membrane without macrotrichia. C distinctly produced, ending about 0.6 times the distance between R_4+5_ and M_1_. Sc short, ending in C but apically weak, not reaching to the base of Rs. Basal cell short, reaching to about one-fourth of the wing length. Vein R_2+3_ relatively short, about ¼ as long as R_4+5_. R-m rather short but distinct. M-fork with a long stem, only slightly shorter than M_1_. Base of M_3+4_ obsolete and slightly approaching Cu. Cu strong, reaching the wing margin. Anal vein short, distally weak, reaching to the level of the base of r-m. Legs entirely dark brown. Coxae as long as height of thorax. Tibiae covered with setulae arranged in dense longitudinal rows. Tibial spurs 1:2:2, about as long as tibial diameter. Relative lengths of tarsomeres 1 to 5 are 1, 0.3, 0.2, 0.15, 0.15. Abdomen dark brown. Terminalia (Figure 2) with narrow gonostylus, distally rounded, slightly longer than gonocoxite, with species-specific structures apically.

**Etymology.** The generic name is derived from “David”, the first name of David Ševčík, the son of the present author, and “*Macrocera*”, a related genus of Keroplatidae.

**Distribution.** Oriental region (Brunei, Taiwan). There are also specimens from Thailand, the Philippines, and Sulawesi, in various collections, currently unavailable to study, indicating a broader distribution of this genus in the region.

#### 3.1.2. *Davimacrocera davidi* sp. nov. (Figure 1b–d and Figure 2a,c,d,f)

LSID: urn:lsid:zoobank.org:act:9B02BB2D-4B60-4CEE-9A58-3E7BD6DE1E6B

**Type material.** Holotype: male, Brunei, Ulu Temburong National Park, Kuala Belalong Field Studies Centre (KBFSC), 4.–10.ii.2013, J. Ševčík and D. Kaspřák leg. (Malaise trap 2), in coll. SMOC. Paratypes: 2 females, the same data as the holotype, in JSL-UOC and UBDC.

**DNA sequences.** DNA sequences of the COI gene marker from the holotype (No. JSOR22) and one female paratype (No. JSOR23) are deposited in the GenBank database under the codes PQ770705 and PQ770706. DNA sequences published from the same locality by Mantič et al. [1] as “genus near *Burmacrocera* sp. 1 (undescribed species)” (specimen No. JSK8) actually belong to an undescribed, closely related species of *Davimacrocera*, which is not treated here because the male specimen is damaged.

**Etymology.** The species is named after David Ševčík, the son of the present author.

**Description.** See the description of the genus (see above). Wing length 2.4–2.5 mm. Male terminalia (Figure 2a) with tergite 9 short, about as long as broad, cerci slightly longer than tergite 9, narrow, and apically rounded. Gonocoxites relatively narrow, fused, with medioventral margin relatively deeply excavated, with excavation reaching almost to half of the length of the gonocoxite. Gonostylus slightly shorter than gonocoxite, narrow, subcylindrical, apically rounded in dorsal view, with a short bidentate projection apicodorsally (Figure 2d).

**Female.** Similar to male, both in color and structural characters, except for terminalia (Figure 2c,f).

**Figure 3:**
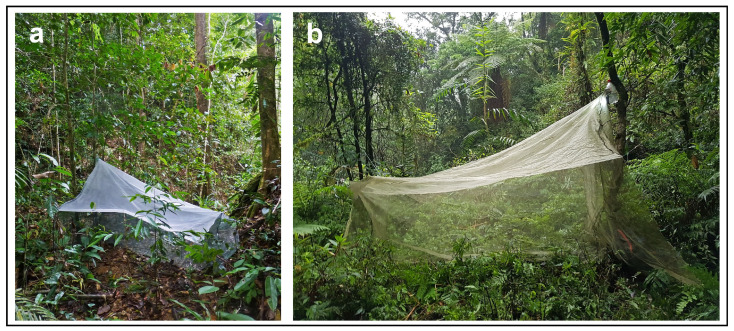
Habitats of the new species, with Malaise traps used to collect the insects. (**a**) Brunei, Ulu Temburong National Park; (**b**) Taiwan, FuShan Botanical Garden. Both photos by J. Ševčík.

**Habitat and phenology.** The type specimens were collected in a primary lowland rainforest (Figure 3a) during winter (in February).

#### 3.1.3. *Davimacrocera taiwanensis* sp. nov. (Figure 1a and Figure 2b,e)

LSID: urn:lsid:zoobank.org:act:90BFCC25-8BBB-4F95-ACC6-86C2FC87581E

**Type material.** Holotype: male, Taiwan (China), FuShan Botanical Garden, 10.v.2018, J. Ševčík leg. (sweep netting in the morning), in coll. NMNS. Paratypes: 4 males, the same data as the holotype except 7.–9.v.2018, J. Ševčík and M. Tkoč leg. (Malaise trap 3), in JSL-UOC, SMOC and NMPC.

**DNA sequences.** DNA sequences of eight gene markers (12S, 16S, 18S, 28S, CAD, COI, cytB, MCS) for this species (male paratype No. JSK8e) were published by Mantič et al. [1] under the provisional name “genus near *Burmacrocera* sp. 2 (undescribed species)”. They are deposited in the GenBank database under the codes MT446493, MT446570, MT446709, MT446798, MT446646, MT446896, MT446978, and MT535463.

**Etymology.** The specific name refers to Taiwan, where the type material was collected.

**Description.** See the description of the genus (see above). Wing length 2.2–2.3 mm. Male terminalia (Figure 2b) with tergite 9 short, about as long as broad, cerci slightly longer than tergite 9, relatively broad, and apically roundly pointed. Gonocoxites relatively broad, fused, with the medioventral margin forming shallow excavation, reaching to about one-third of the length of the gonocoxite. Gonostylus about as long as gonocoxite, narrow, apically pointed in dorsal view, with a dark tooth apicodorsally (Figure 2e). Female unknown.

**Habitat and phenology.** The type specimens were collected in a primary lowland rainforest (Figure 3b) in May 2018. Some specimens were collected early in the morning and in the trap set overnight, suggesting nocturnal activity of this species.

### 3.2. Possible Relationships and Phylogenetic Position of the New Genus

The new genus *Davimacrocera* was included in the dataset of the molecular phylogenetic analysis of Keroplatidae by Mantič et al. [1] under the name “genus near *Burmacrocera*”. Its position was (with high node support) among the genera of Macrocerinae *sensu lato*, comprising the traditional concept of Macrocerinae (tribes Macrocerini and Robsonomyiini), plus the enigmatic genera *Paleoplatyura*, *Schizocyttara* Matile, 1974 and *Sciarokeroplatus* Papp & Ševčík, 2005. The new genus was shown to be a sister group to *Sciarokeroplatus*, but only with moderate support. Both *Sciarokeroplatus* and *Davimacrocera* are small and delicate flies, differing from true macrocerines in some unique characters but also differing from each other. For example, *Sciarokeroplatus* lacks ocelli and the eye-bridge, females have only 9 or 10 flagellomeres [12], and the wing possesses an alula, a unique feature among keroplatids. It also has a longer Sc, longer basal cell, and shorter coxae than in *Davimacrocera*. On the other hand, the overall habitus, shape of antennae in males, and wing venation are very similar in these two genera, possibly allowing the inclusion of *Davimacrocera* in the subfamily Sciarokeroplatinae. This would be, however, premature until a more detailed study of the entire lower Keroplatidae is made, based on both morphology and comprehensive DNA data. At present, I prefer to leave *Davimacrocera* gen. nov. unplaced to a subfamily, as Keroplatidae incertae sedis, within Macrocerinae *sensu lato*, according to Mantič et al. [1].

Similar wing venation and overall habitus can be found also in another Oriental genus, *Microkeroplatus* Ševčík & Papp, 2009, which differs in a number of characters (e.g., specific shape of antennae, lack of R_2+3_, longer tibial spurs, details on the male terminalia); see [13]. In the molecular tree [1], it is placed among the genera of the tribe Keroplatini, subfamily Keroplatinae, indicating that this genus is unrelated to *Davimacrocera* gen. nov. The Neotropical genus *Pseudochetoneura* Ševčík, 2012 and the Oriental *Asiokeroplatus* Ševčík, Mantič & Blagoderov, 2015 also deserve attention in this context [14,15]. Their wing venation shares some similarities with that of *Davimacrocera*, mainly the long stem of the M-fork, but the other body parts, especially the antennae and male terminalia, are quite different. The Afrotropical genus *Micromacrocera* Papp, 2008 shares a very similar wing venation with *Davimacrocera*, differing in the lack of R_3+4_ and in a number of body characters, e.g., a well-developed cerebral sclerite and different structure of the thorax [16].

In the Catalogue of the Oriental Diptera by Colless and Liepa [17], *Burmacrocera minuta* (Senior-White, 1922) is listed from Sri Lanka and the Philippines. This species can possibly belong to *Davimacrocera* gen. nov., although the photos of the holotype (without wings) in the Natural History Museum in London published at the Fungus Gnats Online web page (https://sciaroidea.myspecies.info/taxonomy/term/40719/media, accessed on 23 November 2024) do not provide sufficient details. Judging from the outline of the male terminalia in that photo, this species appears different from *Davimacrocera*. The identity of *B. minuta* may be clarified in the future, as soon as fresh material is collected and its DNA sequenced.

### 3.3. Identity of the Fossil Genus Burmacrocera

The genus *Burmacrocera* was established by Cockerell [7] for the new species, *Burmacrocera petiolata* Cockerell, 1917, based on a single and damaged female specimen preserved in a piece of Burmese amber. It is stated in the original description that the specimen is a male but the swollen abdomen indicates that it is most probably a female (the apical half of the abdomen is missing in the holotype). Since that time, no additional specimen has been reported in the literature, and the identity of this genus remains more or less unclear. In his monograph, Matile [8] placed *Burmacrocera* among the genera of the tribe Orfeliini of the subfamily Keroplatinae, without providing arguments for this action, and this placement was also adopted in the world catalogue of the family [9].

However, the mid-Cretaceous amber of Myanmar, ca. 100 million years old, includes mostly ancient genera of fungus gnats, i.e., the most primitive forms of Keroplatidae, Mycetophilidae, and related families, while the tribe Orfeliini most probably represents the youngest clade of Keroplatidae [1,8], well represented in the Baltic amber (ca 40 MYA) but absent in the Mesozoic deposits, as far as is currently known. I have recently accumulated and studied a private collection of more than 1200 specimens of Sciaroidea in the mid-Cretaceous Burmese amber and I have not found there any single specimen classifiable as Orfeliini. Instead, almost all specimens of Keroplatidae in Burmese amber either belong to Macrocerinae or to other primitive taxa, such as *Paleoplatyura* Meunier, 1899 (see [11]) or the recently described genus *Vladelektra* Evenhuis, 2020, the latter forming a transition to the extant subfamily Lygistorrhininae and classified in the original description as Keroplatidae *incertae sedis* [18].

From the point of view of morphology, the wing venation of *Burmacrocera*, as depicted by Cockerell [7], shows many similarities with Macrocerinae, as well as with *Paleoplatyura*, which is stated already in the original description, and it is also reflected in the generic name. However, the wing venation in Keroplatidae is rather uniform across the family and evidently prone to parallel evolution [1], so other characters must be taken into account too, ideally the structure of the male terminalia. Another characteristic feature of *Burmacrocera* is the thin and tapering antenna, absent in this form among the genera of the subfamily Keroplatinae. Unfortunately, other important characters, such as the structure of the head, the thorax, or the male terminalia, cannot be inferred from the original description because it is based on a single and imperfect female specimen.

**Figure 4:**
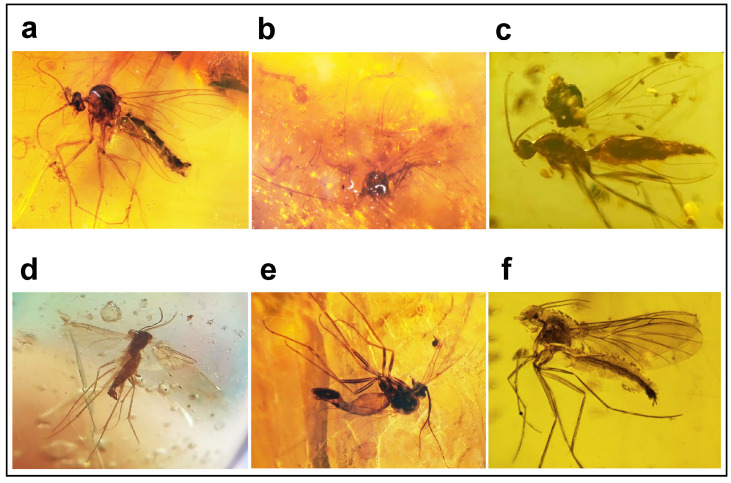
Examples of Macrocerinae *sensu lato* in the Burmese amber collection of the author. (**a**) Male of a genus near *Macrocera* with short antennae; (**b**) male of an undescribed *Macrocera* sp. with a long stem of the M-fork and long antennae; (**c**) female possibly representing *Burmacrocera petiolata*; (**d**) male possibly representing *Burmacrocera* sp. or a related genus; (**e**) male of an undescribed genus of Macrocerinae; (**f**) female of *Macrocera* sp. or a related genus.

There are thus several candidates for being an unknown male of *Burmacrocera*, as well as being the female of *Burmacrocera petiolata*, or a similar species; see Figure 4. All these specimens may be classified as belonging to Macrocerinae or a closely related taxon, differing mostly in the extent of basal constriction of the fork formed by M_3+4_ and Cu, in the length of Sc, and in the presence or absence of Mb. The latter plesiomorphic character state is typical of *Paleoplatyura* and some other ancient genera. The delimitation of Macrocerinae as a subfamily is not an easy task, similar to the delimitation of Keroplatidae as a whole. The most conspicuous character, long antennae, is remarkable but not present in all macrocerine genera. Matile [8] provided the following additional diagnostic characters for Macrocerinae: distinct cerebral sclerite, absence of tibial combs, and basal constriction of the fork of M_3+4_ and Cu. Of these, only wing vein characters can be seen in the original description of *Burmacrocera petiolata*, and this basal constriction is not apparent from the figure. In most specimens of Macrocerinae and related genera in Burmese amber, this constriction is more or less developed, although its visibility and extent are largely dependent on the angle of view.

The situation with *Burmacrocera* illustrates how various problems can arise when a new fossil taxon, genus, or species is described based on a female only, especially in a group like Sciaroidea where specific characters are more pronounced in males. In fossil specimens, association of sexes is practically impossible, except for the rare case when a pair is preserved in copula, in the same amber stone. There is only one published example of a potentially mating pair of Sciaroidea in a piece of Burmese amber [18], and if we accept that the specimens were really mating and not just randomly being close to each other (it is not clear from the photo in [18]), it shows interesting differences in the wing venation and in the structure of antennae between the sexes. If the male and female are not preserved in copula, or at least in the same syninclusion, there is always a possibility that the male and female belong to a different species, especially in genera where several closely related species differ only in minor details on the male terminalia. On the other hand, if only females of a particular species or genus are repeatedly found in various amber pieces, and no males, it may indicate something unusual, e.g., parthenogenesis, as was recently discussed in Zhang et al. [19].

In conclusion, the identity of the Cretaceous genus *Burmacrocera* remains obscure because many similar forms, both males and females, of Macrocerinae *sensu lato* occur in Burmese amber and there is no way to associate them with the female holotype of *Burmacrocera petiolata*.

## Data Availability

The original contributions presented in this study are included in the article. Further inquiries can be directed to the author.
